# Evaluation of traumatic brain injury by optical technique

**DOI:** 10.1186/s12883-015-0465-3

**Published:** 2015-10-14

**Authors:** Bor-Shyh Lin, Che-Chuan Wang, Ming-Hsien Chang, Chung-Ching Chio

**Affiliations:** Institute of Imaging and Biomedical Photonics, National Chiao-Tung University, Tainan, Taiwan; Institute of Photonic System, National Chiao-Tung University, No.901, Zhonghua Rd., Yongkang Dist., Tainan, 710 Taiwan; Department of Medical Research, Chi Mei Medical Center, Tainan, Taiwan; Division of Neurosurgery, Department of Surgery, Chi Mei Medical Center, Tainan, Taiwan; Department of Child Care, Southern Taiwan University of Science and Technology, Tainan, Taiwan

**Keywords:** *Deoxyhemoglobin*, Infarction volume, Near-infrared spectroscopy, Oxy*hemoglobin*, Traumatic brain injury, Triphenyltetrazolium chloride

## Abstract

**Background:**

Traumatic brain injury (TBI), usually due to brain shaking or impact, affects the normal brain function and may lead to severe disability or even death. However, there is paucity of information regarding changes in the physiologic state of humans or animals after brain shaking.

**Methods:**

In this study, near-infrared spectroscopy (NIRS) was used to continuously monitor the concentration change of oxy-hemoglobin (HbO2) and deoxy-hemoglobin (HbR) to understand changes in the physiological state during and after brain shaking. Laser Doppler flowmetry was also used to monitor changes in cerebral blood flow under TBI to supplement the investigation. Triphenyltetrazolium chloride (TTC) staining was used to monitor changes of infarction volume corresponding to different impact strengths.

**Result:**

The experimental results indicated that concentration changes of HbO2 and total-hemoglobin (HbT) were significantly related to the impact strength. The infarction volume was also significantly related to the impact strength.

**Conclusion:**

Therefore, the non-invasive monitoring of concentration changes in *HbO*_*2*_, *HbR*, and *HbT* using NIRS may have a clinical application for the evaluation of TBI.

## Background

Traumatic brain injury (TBI) is often caused by brain shaking or impact. It affects the normal brain function and may lead to disability or death. High-risk groups for TBI are the youth and the elderly, mainly due to the vehicular accidents and falls, respectively. In the clinics, the severity of TBI is usually evaluated using the Glasgow coma scale (GCS) score [1]. For minor TBI, patients may be distracted and suffer short-term amnesia, nonetheless, their nerve function can still fully recover. For moderate TBI (GCS 9–13), patients may become lethargic. For severe TBI (GCS 3–8), patients may be unconscious and cannot open eyes or follow orders [2]. They are at high risk for hypoxemia or brain edema and have 30 % mortality in the first three days [3].

With increasing intracranial pressure (ICP), TBI patients may have ischemia and hypoxia. Under an anoxia metabolism condition, cerebral hematoma and hemorrhage may also occur. Based on the physiologic function of patients, the trauma symptoms that are manifested may not reflect the actual severity of the condition such that abnormal conditions of the patient may be not observed immediately [2] even though intracranial hemorrhage is already happening in the patient’s brain. As this hemorrhage appears gradually in the next few days, it may cause death due to the lack of timely examination and treatment.

Intracranial hemorrhage is the most severe and most difficult to examine. It is also called secondary injury [4], and may be seriously life-threatening because patients with intracranial hemorrhage may initially appear asymptomatic, but suddenly deteriorate at home.

ICP monitoring is usually used to monitor TBI, but it is invasive and may cause brain hemorrhage and infection [5]. Some non-invasive medical instruments, like magnetic resonance imaging (MRI), computed tomography (CT) and positron emission tomography (PET) are used for examining TBI [6, 7] but are widely applied to detect cerebral blood flow and determine oxygen metabolism information of cerebral tissue. However, these medical instruments have enormous cost and poor mobility, which restrict their practical use. Thus, the examination of brain injury is difficult to perform in many instances and in many places. The MRI, without ionizing radiation, is the safest but also the most expensive and its temporal resolution is poorer than CT [8]. Both of PET and CT, which require radioactive substance, are unsuitable for long-term monitoring.

Recently, near infrared spectroscopy (NIRS) were developed and widely applied for cerebral science. The concept of near-infrared spectroscopy was first proposed by Jobsis in 1977 [9]. Near-infrared spectroscopy offers the advantage of non-invasively measuring brain tissue arteriovenous oxygenation via an emitted near-infrared light that penetrates the scalp and underlying brain tissue and detects the absorption of oxyhemoglobin (HbO2) and deoxyhemoglobin (HbR) [10]. As light within the visible spectrum does not penetrate tissue more than approximately 1 cm, wavelengths in the near-infrared region between 650 and 900 nm are used, permitting deeper penetration [11]. In contrast to pulse oximetry, in which only the arterial component is considered, NIRS captures an average of the arterial, capillary, and venous compartments. By using such light to penetrate through the brain and monitor their variation of relative optical transparency, the concentration changes of HbO2 and HbR, which is relation to cerebral blood volume and oxygen metabolism, can be calculated. Thus, near-infrared spectroscopy can be applied to detect focal cerebral ischemia [12–14], hemorrhage [15–17], newborn infant hypoxia [18], and post-injury cognitive functions [19].

The present study investigated the change of HbO2 and HbR concentrations during and after TBI using an NIRS system. After TBI, the brain tissue was injured and cause the change of the change HbO2 and HbR. The change of HbO2 and HbR concentrations are related to the change of cerebral blood volume (CBV), but not the change of blood flow. In order to obtain more detail information to understand the change of physiologic state under different impact levels, laser Doppler flowmetry (LDF) is also used to monitor the change of cerebral blood flow (CBF). Finally, Triphenyltetrazolium chloride (TTC) staining was used to determine the relationship between changes of infarction volume and impact strength. From the relationship between the change of HbO2 and HbR concentrations, infarction volume, and impact levels, the NIRS system can be considered as an alternative tool to provide useful information of TBI via simple, portable and non-invasive measurement.

## Methods

### Near-infrared spectroscopy system

A continuous wave-based NIRS system was assembled to monitor changes in the relative HbO2 and HbR concentrations under traumatic brain injury. The sampling rate of the NIRS system was set to 25 Hz. The optical probe of the NIRS system contains two pairs of dual light-emitting diodes (SMT735/850, EPITEX, Japan) and photodiodes (PD15-22C/TR8, EVERLIGHT, Taiwan) that were used to supply the red and infrared light sources, and transfer the intensity of diffusely reflective light into current or voltage, respectively. Here, the light sources with 735 and 850 nm wavelength were used to provide better penetration ability. When light was emitted onto the biological tissue, only some light penetrate through the tissue due to the scattering and absorbing properties of the different structures in the tissue. Thus, the penetrating light usually carried the physiological information of the tissue.

In general, the penetrating depth of the red and infrared light is about a half of the distance between the light source and the detector [20, 21]. The specific area for monitoring changes in HbO2 and HbR was determined by using this rule. In this study, the distance between the light source and the detector is set to 16 mm. As such, Δ[*HbT*] denotes the concentration change in total-hemoglobin and was calculated by the formula:

1 $$ \Delta \left[HbT\right]=\Delta \left[Hb{O}_2\right]+\Delta \left[HbR\right] $$

where Δ[*HbO*_*2*_] and Δ[*HbR*] denote the change in HbO2 and HbR concentration respectively.

### Animals preparation

Adult male Sprague Dawley rats (weight, 375 ± 25 g) were prepared. All rats were kept under a 12-hour light/ dark cycle, and allowed free access to food and water. All of the experimental procedures conformed to the guidelines of National Institute of Health of Taiwan and were approved by the Animal Care and Use Committee of Chi Mei Medical Center to minimize discomfort to animals during surgery and recovery periods. The rats were randomly assigned to two groups : Group 1 (*n* = 22) were monitored under the fluid percussion injury experiment with different impacts (1.6, 1.8, 2.0, 2.2 and 2.4 a.t.m.) using our NIRS system. The number of the rats for 1.6, 1.8, 2.0, 2.2, and 2.4 a.t.m. are 4, 4, 6, 4, and 4 respectively. Group 2 (*n* = 4) were monitored by using LDF.

All of the rats were anesthetized with sodium pentothal (25 mg/kg, i.p.; Sigma Chemical Co, St Louis, MO) and a mixture containing ketamine (44 mg/kg, i.m.; Nan Kuang Pharmaceutical, Tainan, Taiwan), atropine (0.02633 mg/kg, i.m.; Sintong Chemical Industrial Co, Ltd, Taoyuan, Taiwan), and xylazine (6.77 mg /kg, i.m.; Bayer, Leverkusen, Germany). All of the rats were sacrificed on the 3^rd^ post-surgery day and rat brain slices were stained by TTC solution.

### Experiment design for traumatic brain injury

**Fig. 1:**
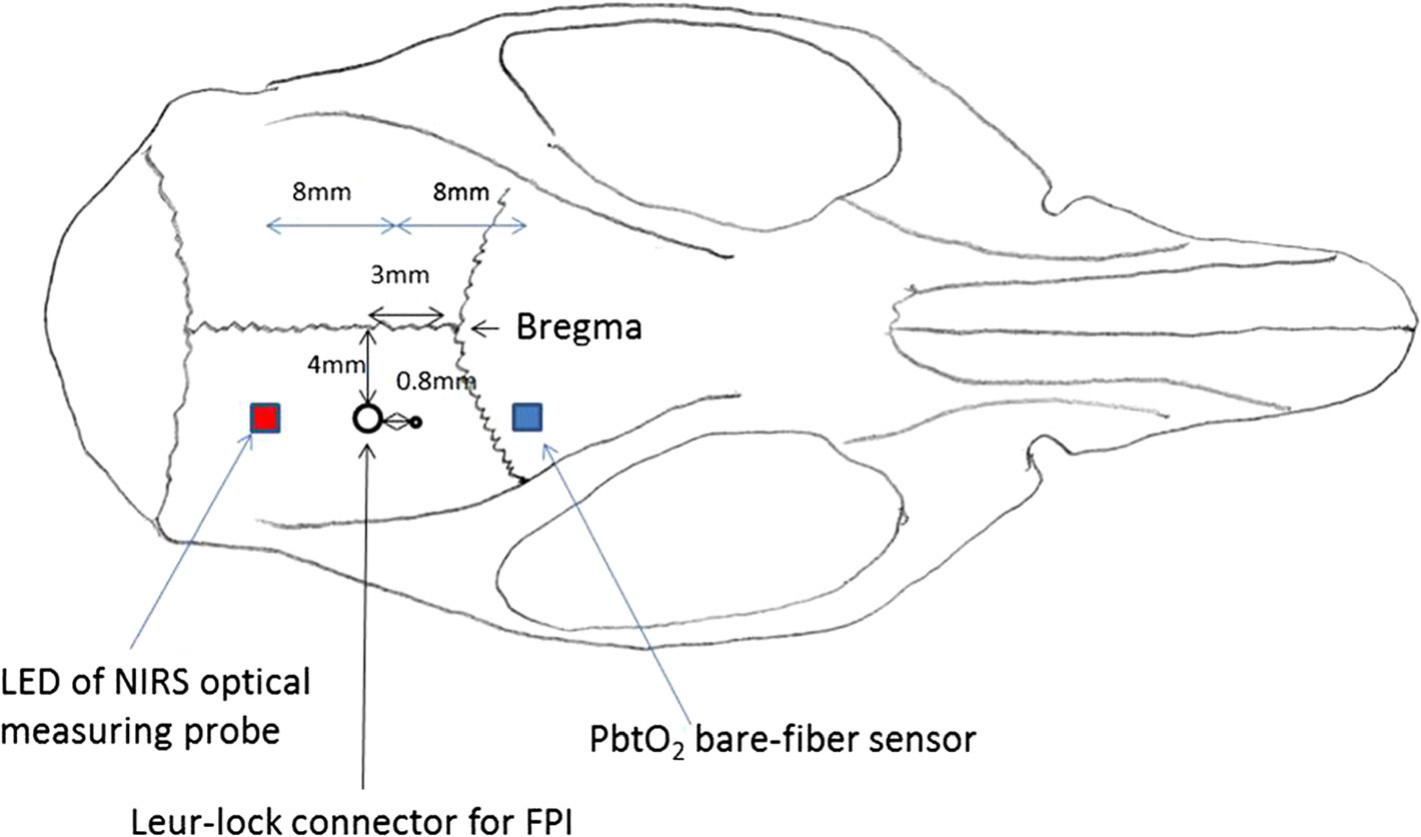
Monitoring positions for NIRS were at striatum region of rat brain, anterior-posterior −0.5 mm and lateral +3.5 mm from bregma, under top of rat brain 8 mm (includes 1 mm rat skull). Distance between dual light-emitting diodes and photodiodes was set to 16 mm. And catheter of LDF was inserted on the top of rat brain approximately 5–6 mm

The fluid percussion injury (FPI) experiment was used as the rat model for the traumatic brain injury [22]. Before beginning the FPI experiment, the rat was anesthetized and its head placed in a stereotaxic frame, with ear bars ears inserted to the ears to tight the head. A rectal temperature probe was inserted into the colon and attached to the thermostatic controller that powered the heating pad to maintain the rat core temperature at 37 °C. The fur on the rat head was then trimmed and the scalp was incised sagittally. In order to attack the rat brain directly, a hole on the skull was drilled to expose the brain. The hole was placed −3 mm anterior-posterior and +4 mm lateral to the bregma. A leur-lock connector was cemented to the craniotomy with cyano-acrylic adhesive and dental acrylic. The other side of the connector connected to a sealed and fluid-filled reservoir. A pendulum struck the reservoir to generate a fluid wave to attack the rat brain and form fluid percussion injury (Fig. 1).

For Group 1, the monitoring positions for NIRS were at striatum region of rat brain, anterior-posterior −0.5 mm and lateral +3.5 mm from bregma, under top of rat brain 8 mm (includes 1 mm rat skull). For Group 2, a LDF was used to monitor cerebral blood flow in the injured region. The LDF probe was placed −0.8 mm anterior-posterior and +4 mm lateral to the bregma, and was installed on the stereotaxic frame (Fig. 1). Before beginning the FPI experiment, changes in 30-second HbO2 and HbR were recorded as baseline data. The FPI experiment was then conducted and after, the rat was removed from the FPI device. Since tranisent apnea occurred after FPI, a respiratory treatment procedure by withdrawing the rat’s tongue out of mouth was performed immediately after FPI for protect the airway patent [23]. The treatment procedure was completed within 5–10 min.

**Fig. 2:**
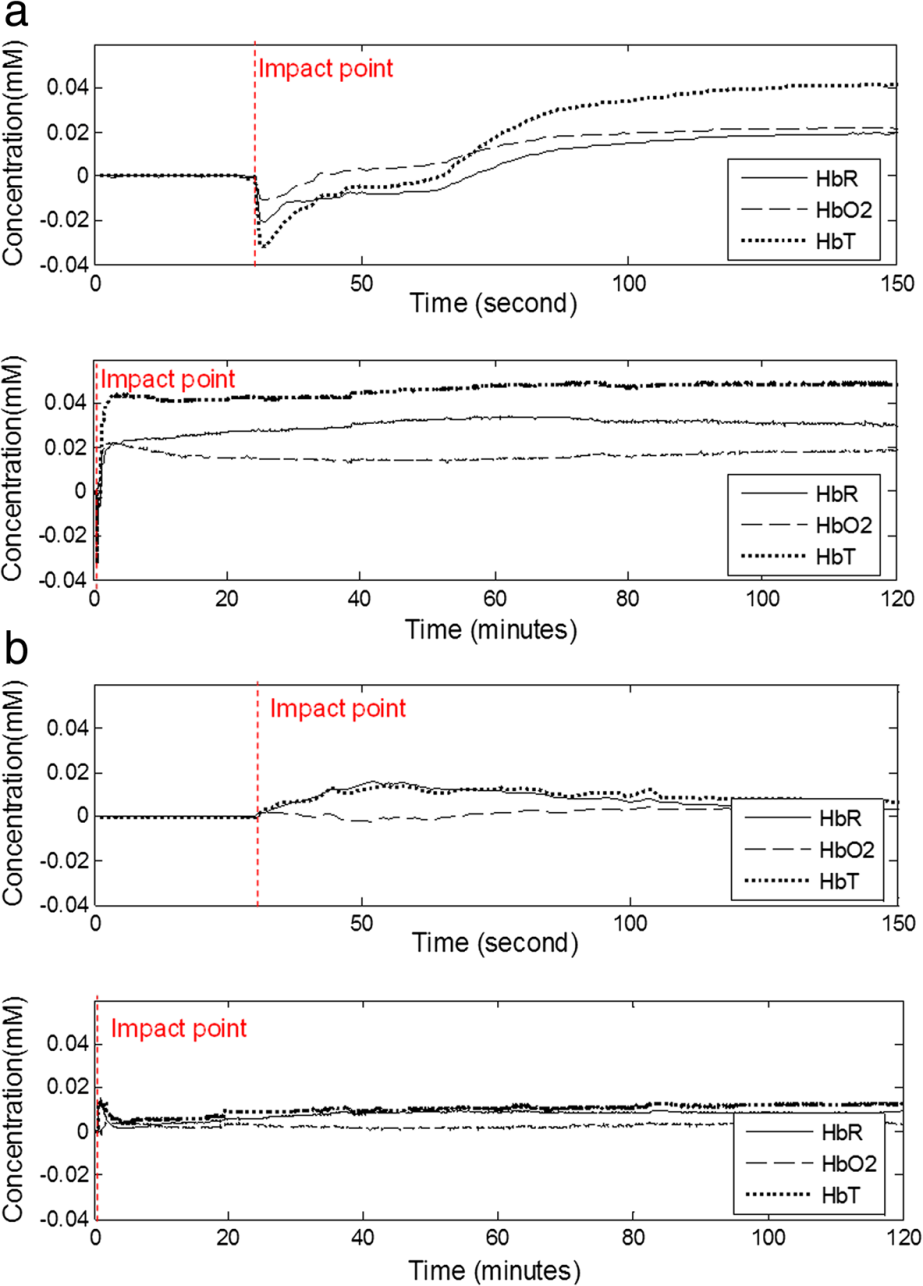
Temporal profiles of Δ[*HbO*_*2*_], Δ[*HbR*], and Δ[*HbT*] in (**a**) ipsilateral side and (**b**) contralateral side during and after FPI experiment for 1.6 a.t.m. Δ[HbR], and Δ[HbT] dropped immediately upon impact in ipsilateral side. After the procedure of respiratory treatment, the rebreathing from apnea resulted in that all of Δ[HbO2], Δ[HbR] , and Δ[HbT] increased

The temporal profiles of Δ[HbO2], Δ[HbR], and Δ[HbT] were obtained from the ipsilateral and contralateral sides (Fig. 2a and Fig. 2b). In order to investigate the status of brain injury during and after the FPI experiment, concentration changes in HbO2 and HbR during the FPI experiment, and the long-term concentration changes in HbO2 and HbR after the FPI experiment were determined. The HbO2 and HbR changes of the brain-injured rat were monitored for 2 h continuously. After all of the experimental procedures, the connector and the acrylic on the rat head were removed and the incisions on the rat were sutured.

### Statistical analysis

Physiologic data were analyzed with repeated-measure ANOVA for differences between time points and groups. Significance was set at *p* < 0.05. Data were expressed as mean ± standard deviation (SD) of the mean. Here, Matlab was used to perform repeated-measure ANOVA.

## Results

### Variation of cerebral blood oxygenation under traumatic brain injury

The randomly selected experimental result for the temporal profiles of Δ[HbO2], Δ[HbR], and Δ[HbT] in ipsilateral side and contralateral side during and after FPI experiment, with the impact strength set to 1.6 a.t.m., were shown in Fig. 2a and Fig. 2b, respectively. Both of HbO2 and HbR concentrations dropped immediately when the impact occurred (Fig. 2a). Both of HbO2 and HbR concentrations increased during the period of respiration treatment and became larger than their baselines data after the treatment. During and after the respiration treatment, *HbT* concentration also increased. After the respiration treatment, Δ[*HbR*] became obviously larger than Δ[*HbO*_*2*_]. On the contralateral side (Fig. 2b), concentration variations in *HbO*_*2*_ and *HbR* during and after FPI were also slightly affected by the impact.

### Effect of impact strength on variation of cerebral blood oxygenation

**Fig. 3:**
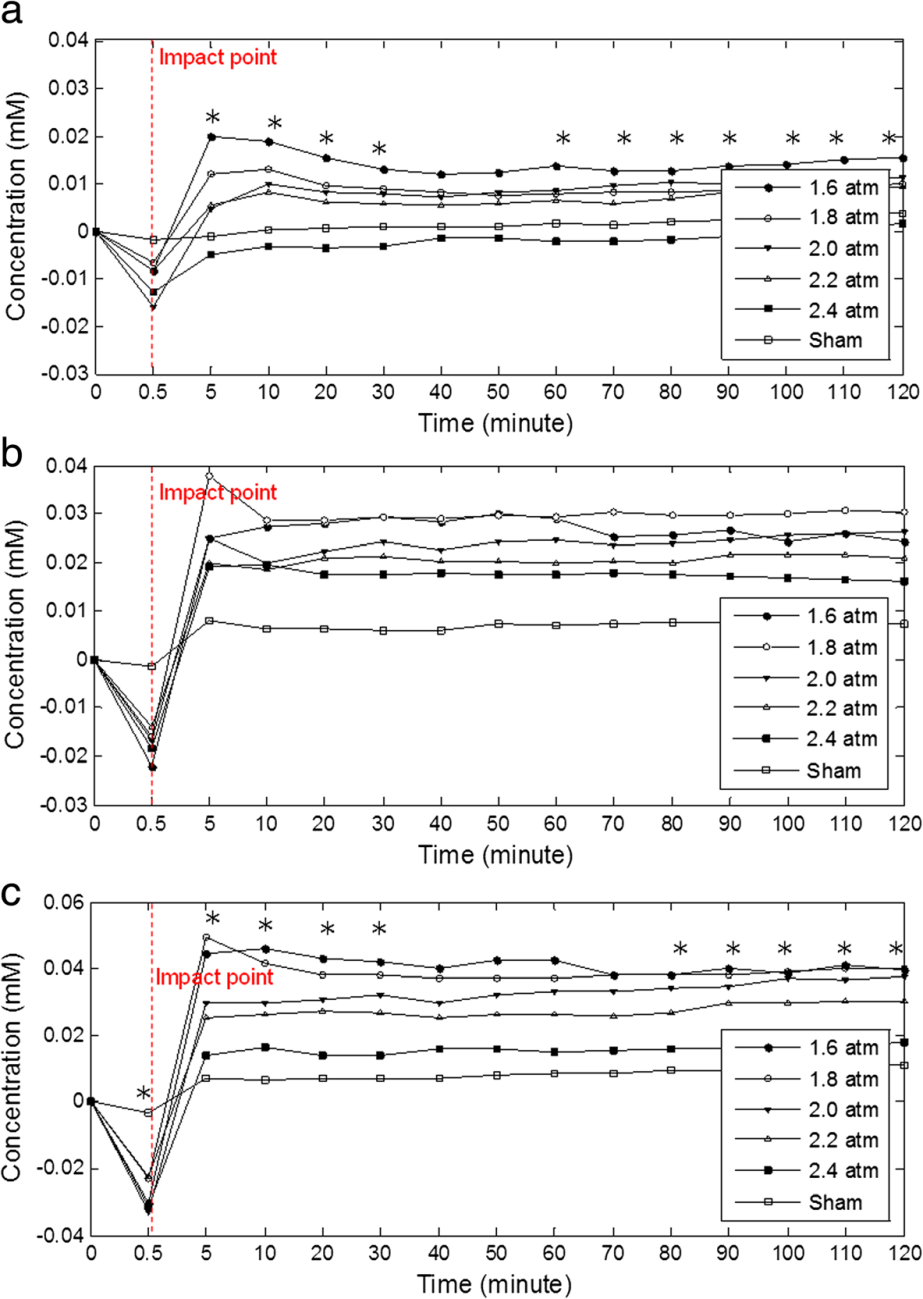
Time courses of (**a**) Δ[*HbO*_*2*_], (**b**) Δ[*HbR*], and (**c**) Δ[*HbT*] corresponding to different impact strength groups (* means significance)

The effect of the impact strength on variation of cerebral blood oxygenation was investigated. The time courses of the averages of Δ[*HbO*_*2*_], Δ[*HbR*], and Δ[*HbT*] corresponding to different impact strength groups were shown in Fig. 3a to c, respectively.

Repeated-measure ANOVA for differences between groups at different time points was used to analyze experimental data, with impact strengths set to 1.6, 1.8, 2.0, 2.2, and 2.4 a.t.m. Results showed that concentration variation tendencies of *HbO*_*2*_, *HbR*, and *HbT* corresponding to different impact strengths were similar. All three measures dropped immediately upon impact and the concentration changes in *HbO*_*2*_ and *HbT* corresponding to different impact strengths were significant. However, the relationships between the impact strengths and the concentration changes in *HbO*_*2*_, *HbR*, and *HbT* on the ipsilateral side at the moment of impact were not linear and were significantly different from changes in the contralateral side. Subsequently, the common tendencies of concentration changes in *HbO*_*2*_, *HbR*, and *HbT* for different impact strengths increased and then became stable gradually.

Moreover, the concentrations of *HbO*_*2*_, *HbR*, and *HbT* after impact depend significantly on the impact strength. After the impact, the concentrations of *HbO*_*2*_, *HbR*, and *HbT* decreased with the increasing impact strength.

## Discussion

When the impact occurred, the outer pressure from the impact effecting a contraction of the cerebral vasculature, and this also resulted in that all of *HbO*_*2*_, *HbR*, and HbT concentrations dropped immediately upon impact (Fig. 2a). After the procedure of respiratory treatment, the rebreathing from apnea resulted in that all of *HbO*_*2*_, *HbR*, and HbT concentrations increased. Moreover, Δ[*HbT*] is related to cerebral blood volume [24]. The increase of HbT concentration after respiratory treatment can be explained by vascular dilation and perfusion phenomenon, which may also cause edema. And after respiratory treatment, the Δ[*HbR*] is obviously larger than the Δ[*HbO*_*2*_], which can be explained by the impaired cells in the cerebral tissue requiring more oxygen to promote cell metabolism. On the contralateral side, concentration variations in HbO2 and HbR are also slightly affected by the impact. The results reveal that impact pressure can be transmitted to the contralateral side and may also cause slight brain injury and then alter neurotransmitter activities on the contralateral side [25].

Subsequently, the common tendencies of concentration changes of HbO_2_, HbR, and HbT for different impact strengths increase and then become gradually stable (Fig. 3). This is due to the vascular expansion after the impact causes an increase in CBV. Simultaneously, HbO_2_ releases oxygen to support damaged brain cells and this leads to the increase in *HbR* concentration and the decrease in *HbO*_*2*_ concentration. Therefore, Δ[*HbR*] is obviously larger than Δ[*HbO*_*2*_]. On the contralateral side, concentration variations in *HbO*_*2*_ and *HbR* are also slightly affected by the impact. The slight pressure of the impact still transmitted to the contralateral side, and may also cause slight brain injury on the contralateral side.

After the impact, concentrations of *HbO*_*2*_, *HbR*, and *HbT* decreased with increasing impact strength. This is because increasing impact strength induces more severe edema that obstructs blood flow to the brain. The impact strength of 2.4 a.t.m. is set as the highest strength in the FPI experiment and is usually regarded as severe TBI [26]. After suffering the 2.4 a.t.m. impact, concentrations of *HbR* and *HbT* increased and became larger than the baseline, whereas that of *HbO*_*2*_ concentration became lower than baseline value. Although *HbT* concentration still increases after the 2.4 a.t.m. impact, the increase in *HbT* concentration is significantly smaller than those of other impact strengths. After the 2.4 a.t.m. impact , the increase in *HbO*_*2*_ concentration cannot provide sufficient oxygen to satisfy the requirement of metabolism.

**Fig. 4:**
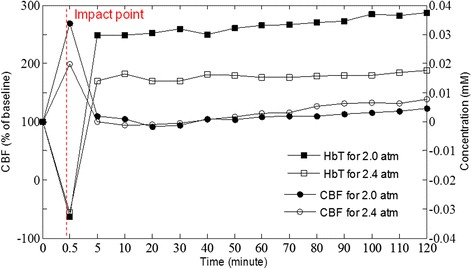
Averaged temporal profiles of CBF and HbT in ipsilateral side during and after FPI experiment for 2.0 a.t.m. and 2.4 a.t.m [27]. (Acknowledge the source of this finger which published in The proceedings of 35^th^ Annual International Conference of the IEEE Engineering in Medicine and Biology Society 2013, 2013: 2412–2414)

To understand the change in physiological state under TBI, laser Doppler flowmetry was used to monitor the change CBF. Variation in CBF during and after the FPI experiment (Fig. 4) [27] showed that CBF increased immediately at the moment of impact and then decreased rapidly. After respiration treatment, CBF increased gradually. Compared with Δ[*HbT*], the tendency of CBF is different from that of cerebral blood volume upon impact. This may be because the vessels in the injured region are constricted, resulting in the increased local CBF but decreased CBV. Vessel constriction causes increased blood flow velocity.

After the impact, the value of CBF is reduced and becomes even smaller than the baseline, but the concentration change of *HbT* tends to increase. This may be due to vessel dilation after the impact. Although the blood flow velocity decreased, cerebral blood volume still increased. Finally, CBF increased gradually, fitting the phenomenon of increasing HbT concentration (Fig. 3C).

**Fig. 5:**
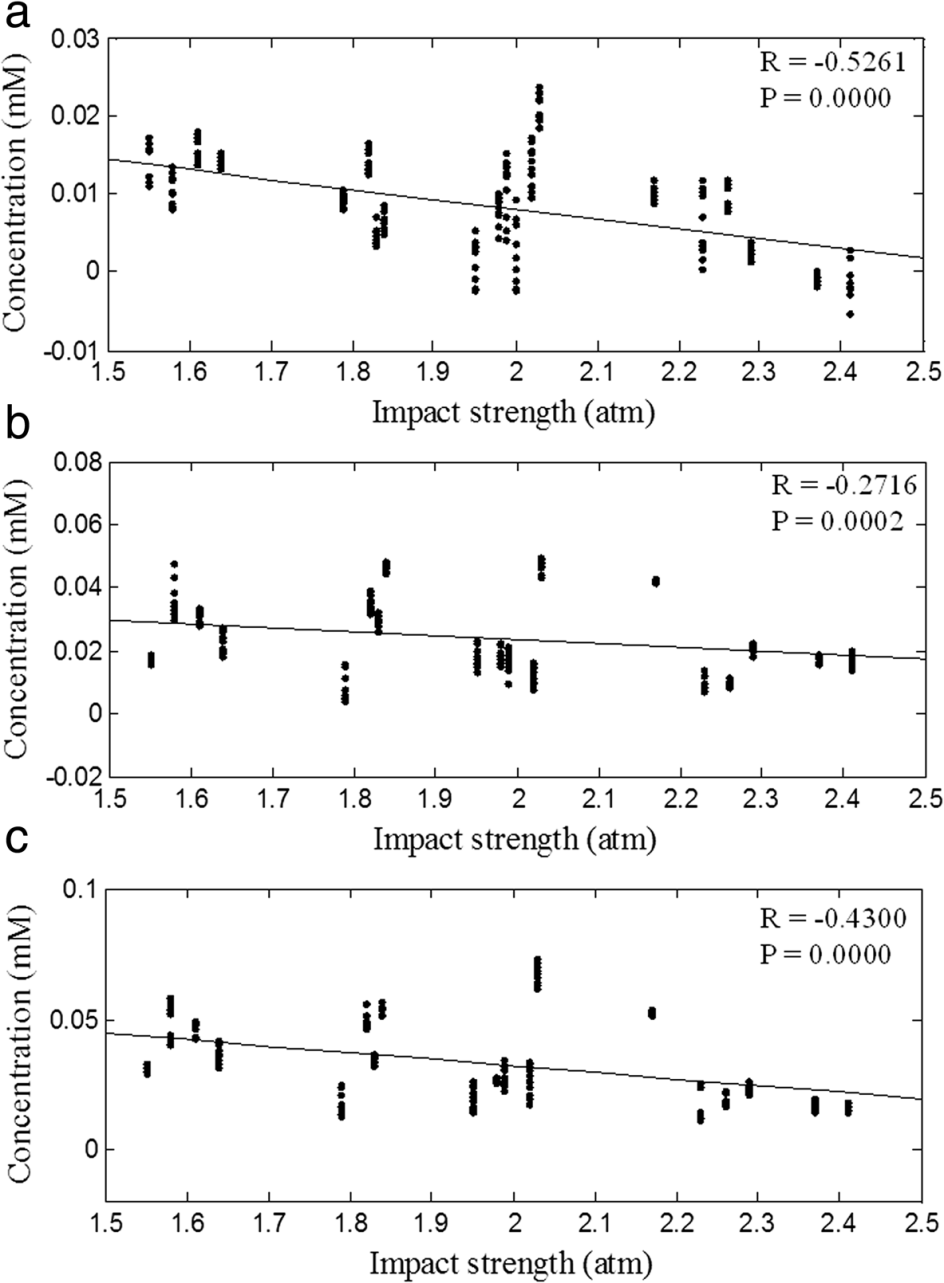
Correlation between different impact strength groups and (**a**) Δ[*HbO*_*2*_], (**b**) Δ[*HbR*], and (**c**) Δ[*HbT*]. correlation between different impact strength groups and Δ[HbO2], Δ[HbR], and Δ[HbT] are −0.5261 (*p* = 0.00002), −0.2716 (*p* = 0.0002), and −0.4300 (*p* = 0.00003)

Correlations between impact strength groups and Δ[*HbO*_*2*_], Δ[*HbR*], and Δ[*HbT*] are shown in Fig. 5a to 5C, respectively. The Δ[*HbO*_*2*_], Δ[*HbR*], and Δ[*HbT*] values from 30 min to 120 min after the impact show that the correlation between impact strength and Δ[*HbO*_*2*_], Δ[*HbR*], and Δ[*HbT*] are −0.5261 (*p* = 0.0000), −0.2716 (*p* = 0.0002), and −0.4300 (*p* = 0.0000). In the correlation with impact strength, Δ[*HbO*_*2*_] and Δ[*HbT*] are higher than Δ[*HbR*].

**Fig. 6:**
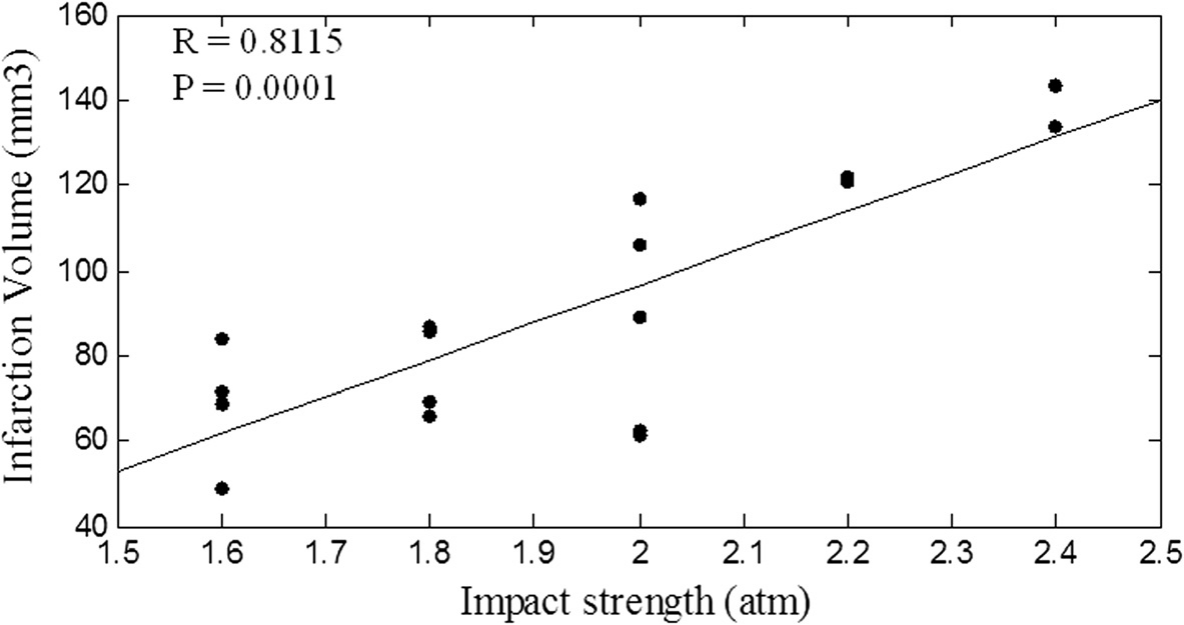
Correlation between impact strength and infarction volume. The correlation between the different impact strength groups and infarction volume is high (*r* = 0.8115) and significant (*p* = 0.0001)

The correlation between the different impact strength groups and infarction volume (Fig. 6) is high (*r* = 0.8115) and significant (*p* = 0.0001). From the above results, it shows that the infarction volume is proportional to the impact strength.

## Conclusions

Investigating concentration changes of *HbO*_*2*_, *HbR*, and *HbT* during and after TBI shows that the concentration change of *HbO*_*2*_, *HbR*, and *HbT* decreased immediately upon impact, and increased gradually after respiration treatment. Compared to the change of CBF are measured by LDF, the change in vessels and edema states during and after TBI can be clearly understood. The concentration changes of HbO_2_ and HbT after impact significantly depend on impact strength. The concentration change of HbO2, HbR, and Hbt after impact decrease with increasing impact strength. Moreover, the infarction volume is also significantly proportional to the impact strength. Therefore, the non-invasive monitoring of concentration changes in HbO_2_, HbR, and HbT using NIRS may have a clinical application for the evaluation of TBI.

## Abbreviations

TBI: Traumatic brain injury
NIRS: near-infrared spectroscopy
HbO2: oxy-hemoglobin
HbR: deoxy-hemoglobin
TTC: Triphenyltetrazolium chloride
GCS: Glasgow coma scale
ICP: intracranial pressure
MRI: magnetic resonance imaging
CT: computed tomography
PET: positron emission tomography
LDF: Laser Doppler flowmetry
CBF: cerebral blood flow
FPI: fluid percussion injury
CBV: cerebral blood volume
